# Synthesis and Electrorheological Response of Graphene Oxide/Polydiphenylamine Microsheet Composite Particles

**DOI:** 10.3390/polym12091984

**Published:** 2020-08-31

**Authors:** Chun Yan Gao, Min Hwan Kim, Hyoung-Joon Jin, Hyoung Jin Choi

**Affiliations:** Department of Polymer Science and Engineering, Inha University, Incheon 22212, Korea; 22151729@inha.edu (C.Y.G.); minhwannn@gmail.com (M.H.K.); hjjin@inha.ac.kr (H.-J.J.)

**Keywords:** conducting polymer, graphene oxide, polydiphenylamine, electrorheological

## Abstract

Conducting graphene oxide/polydiphenylamine (GO/PDPA) microsheet nanocomposite particles were fabricated via in-situ oxidative polymerization using diphenylamine in the presence of GO. The morphological structures and dimensions of the fabricated GO/PDPA composites were evaluated using transmission electron microscopy and scanning electron microscopy. Electrorheological (ER) responses and creep behaviors of an ER fluid consisting of the GO/PDPA composites when suspended in silicone oil were evaluated using a rotational rheometer under input electric field. Three different types of yield stresses were examined along with dielectric analysis, demonstrating their actively tunable ER behaviors.

## 1. Introduction

Intelligent and smart fluids have drawn widespread attentions from industries and academia because of their sensitivities to applied stimuli, including electric and magnetic fields, pressure, temperature, and light [1,2]. Among such fluids, electrorheological (ER) fluids, which are usually colloidal suspensions comprising polarizable or semiconductive solid materials dispersed in insulating phase media, have been one of the most investigated smart materials [3]. Their electrical responsive properties include agile and reversible transformations between a liquid- and a solid (or chain)-like phase with and without an input electrical field, respectively, in a period of the order of milliseconds [4,5]. Without an electric field, the particles in the suspension randomly dispersed in insulating liquids behave as Newtonian fluids. In contrast, by applying an electric field, the randomly suspended particles instantaneously arrange in a column-like form following the field direction owing to dipole–dipole interactions between the polarized particles. The aforementioned changes in the ER suspension state lead to significant differences in the rheological behaviors compared to those of the smart fluid without the input electrical field. The increase in the shear viscosities, yield stresses, and viscoelastic moduli depends on the magnitude of the external electrical field strength; in this case, the ER fluids generally behave similar to a Bingham fluid or other types of non-Newtonian fluids. Particularly, the tightly arranged chain-like structures can relax into an irregular liquid phase once the external electric field is removed; this process is repeatable, manageable, reversible, and instantaneous. Owing to these valuable properties, they have numerous industrial applications in various fields, including those involving smart robots, anti-vibration systems, actuators, and food processing [6,7,8].

Over the past several decades, numerous smart ER materials, including cellulose, oxide minerals, carbon nanotube, graphene, graphene oxide (GO), ionic liquids, and conducting and semiconducting polymers and their composites, have been studied [9,10,11,12]. Among these ER materials, GO is highly promising owing to its characteristic structure and properties such as electric conductivity, flexibility, large surface area, thermal stability, and good hydrophilic behavior. The oxygen associated parts on the surface of GO increase its dispersion stabilities in various solvents in addition to increasing the possibility of the formation of composites between GO and other substances. In contrast with neat graphene, the poorly conductive oxidation state of GO (10^−6^ S/cm) is suitable for its ER applications. In their pioneering report, Zhang et al. [13] presented the synthesis of pure GO and the ER characteristics of GO when it is suspended in silicone oil. The ER performance of GO is lower than that of other conducting polymers, probably due to its flexible property even without an electrical field. Moreover, uniform-size core–shell-typed polystyrene (PS)/GO composite particles produced via the π–π interaction between the surfaces of the PS spheres and GO sheets, and their ER behaviors under an input electrical field, have been investigated [14]. Lee et al. [15] have reported the fabrication of Fe_3_O_4_/SiO_2_/GO spherical composites having dual stimulus responses in an electric and magnetic field-based smart fluid. Owing to the SiO_2_ and magnetic Fe_3_O_4_, the yield stress and modulus of the smart fluid under the electrical field were considerably smaller than those under the magnetic field. Other GO-based composites, such as polyaniline (PANI)/GO [16], poly(p-phenylenediamine)/GO [17], and poly(methyl methacrylate)/GO composites, were also studied [18]. For the high-conductivity PANI/GO composite, a dedoping step must be performed before the fabrication of the ER fluid. Conductive polymers with π-conjugated structures, such as PANI, have attracted considerable attention with regard to ER materials owing to their inherent polarizations, easy changes in conductivities, and low densities. Their conductivities can be adjusted through doping or dedoping to achieve a suitable semiconducting range to avoid electrical short circuits under an external electric field. Recently, we used polydiphenylamine (PDPA) to develop an ER material owing to its simple synthesis, low density of 0.96 g/cm^3^ [19], good optical properties, eco-friendliness, and good environmental stability [20]. In comparison to PANI, a typical conducting polymer used in ER fluids, PDPA provides a higher thermal stability, higher compatibility with organic solvents, and lower conductivity. Thus, it can be used directly for designing ER fluids without performing dedoping, which is required for most conductive polymers [21].

In this study, GO/PDPA microsheet particles were fabricated, with a density 1.45 g/cm^3^ lower than that of neat GO (1.78 g/cm^3^), via in-situ oxidative polymerization [13]. The numerous π bonds on the GO surface serve as a crucial factor for the occurrence of polymerization on the GO surface. Moreover, the ER responses and creep properties of a semiconducting GO/PDPA-composite-based ER suspension were tested.

## 2. Experimental

### 2.1. Materials and Synthesis

Graphite (99.99%, powder size < 45 μm, Sigma-Aldrich, St. Louis, Missouri, USA) was adopted as a raw material for the synthesis of GO. Potassium permanganate (KMnO_4_, Sigma-Aldrich, St. Louis, MO, USA), sodium nitrate (NaNO_3_, Junsei Chem., Tokyo, Japan), and sulfuric acid (98%, DC Chem., Seoul, Korea) were employed, as purchased, without further purification treatment. Diphenylamine (DPA) (99%, Sigma-Aldrich, St. Louis, MO, USA) and ammonium persulfate (APS) (98%, Daejung Chem., Shiheung, Korea) were adopted as a monomer and initiator for the chemical oxidation polymerization, respectively.

GO sheets were synthesized with graphite using the modified Hummers process [22]. NaNO_3_ (1.0 g) was initially dispersed in a concentrated sulfuric acid solution (140 mL) under continuous stirring at 450 rpm until the solution cooled to room temperature. Graphite (2 g) and KMnO_4_ (6 g) were then added into the sulfonic acid solution. After stirring for 30 min, the solution was sonicated for 15 min and stirred continuously for 2 h at 150 rpm. Subsequently, the suspension was slowly added to deionized water (350 mL). Finally, a 30% hydrogen peroxide solution (300 mL) was rapidly introduced. Stirring was continued for 0.5 h to obtain the final GO solution. All of the aforementioned steps, except the sonication process, were carried out in a ventilated facility. The mixture was washed using deionized water via centrifugation and freeze-dried to obtain purified GO.

**Scheme 1:**
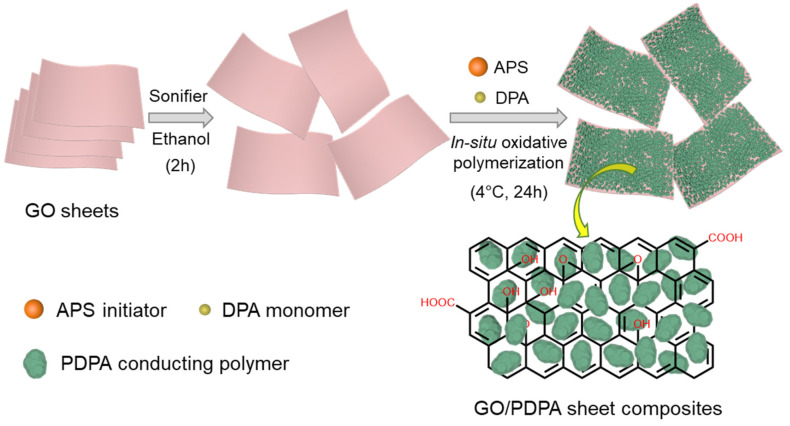
Schematic diagram of the synthetic process of graphene oxide/polydiphenylamine (GO/PDPA) microsheet composites.

GO/PDPA microsheet composites were fabricated via in-situ oxidative polymerization, as shown in Scheme 1. The monomer DPA (0.025 mol) was dispersed in ethanol (100 mL) under magnetic stirring for 20 min at 4 °C. The solution of GO (0.37 g) dispersed in ethanol (150 mL) pretreated using a sonicator for 2 h was then added. The initiator APS (0.025 mol), in deionized water (50 mL), was introduced to the above-mentioned suspension. The polymerization was allowed to continue for a day at 4 °C. The product was cleaned with ethanol and deionized water several times, and then dried in vacuum. Through the polymerization-promoting π–π interactions between the surfaces of the GO sheets and the DPA monomer, PDPA was uniformly attached to the surface of GO. The ER fluid (10 vol.%) was prepared by suspending the GO/PDPA microsheet naocomposite particles in silicone oil (100 cS) through ultrasonication.

On the other hand, it can be also noted that the reduced GO/PDPA has been synthesized via the electrochemical polymerization of DPA on reduced GO, along with its various optical characterizations [23], while Muthusankar et al. [24] studied electrochemical behaviors of PDPA/phosphotungstic/GO hybrid.

### 2.2. Characterization

The morphological structures and sizes of the neat GO sheets and GO/PDPA microsheet composites were evaluated using transmission electron microscopy (TEM) (CM-220, Phillips, Amsterdam, The Netherlands) and high-resolution scanning electron microscopy (HRSEM) (SU-8010, Hitachi, Tokyo, Japan). The chemical structures were evaluated using the infrared spectra of mixtures of KBr pellets and the samples via Fourier-transform infrared (FTIR) spectroscopy (VERTEX 80 V, Bruker, Ettlingen, Germany). X-ray diffraction (XRD) (DMAX-2500, Rigaku, Tokyo, Japan) was performed to characterize the crystalline structures of the neat GO and GO/PDPA composites. The electrical conductivities of pure GO sheets and GO/PDPA composites were tested by a low resistivity meter (Loresta-GP MCP-T610, Mitsubishi chem., Tokyo, Japan) equipped with a square probe (MCP-TPQPP, Mitsubishi chem., Tokyo, Japan). The alignment of the GO/PDPA-based ER suspension was tested directly via optical microscopy (OM) (BX51, Olympus, NY, USA) at a direct-current voltage of 300 V. The dielectric properties of the GO/PDPA-composite-based ER fluid were analyzed using an inductance–capacitance–resistance (LCR) meter (HP 4284A Precision, Agilent, Santa Clara, CA, USA) with an HP16452A liquid test fixture. The ER characteristics of the conducting composite-based ER fluid were measured using a rotational rheometer (MCR 300, Anton Paar-Physica, Graz, Austria) comprising a direct-current high-voltage generator (HCP 14-12 500 MOD, FuG Elektronik GmbH, Rosenheim, Germany) with a Couette-type cell (CC17).

## 3. Results and Discussion

### 3.1. Material Property

**Figure 1:**
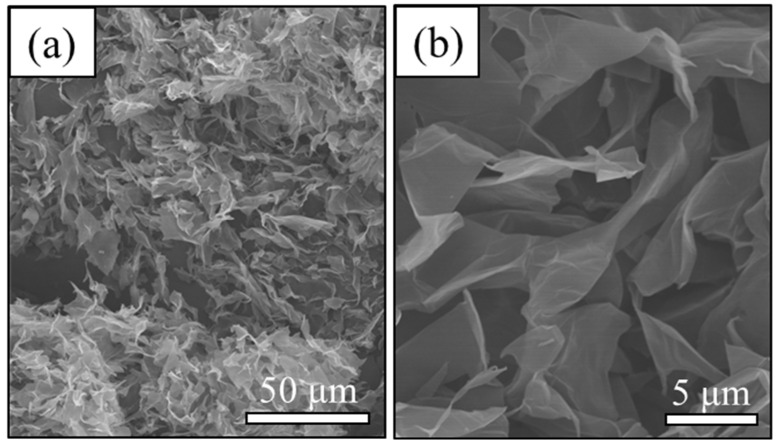
HRSEM images of pure GO microsheets.

**Figure 2:**
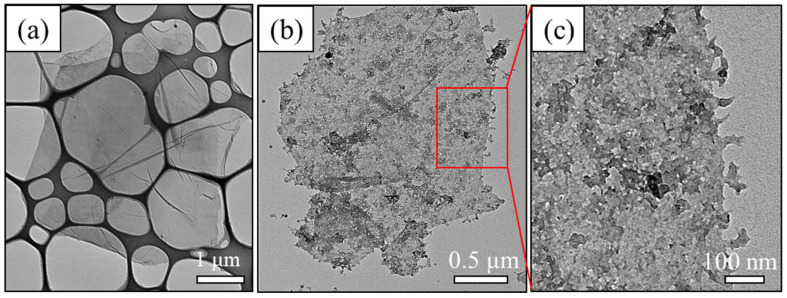
TEM images of (**a**) pure GO and (**b**,**c**) GO/PDPA microsheet composites.

Figure 1 and Figure 2 show the surface morphologies and dimensions of the microscale pure GO and GO/PDPA composite platelets. The HRSEM images show the pure GO sheets of a few micron grades (Figure 1). Their surfaces are very smooth. In the GO/PDPA sheet composite, PDPA is uniformly polymerized on the GO surface, yielding a rougher appearance (Figure 2) than that of the pure GO sheet. The sizes of the fabricated sheets are slightly smaller when determined using the TEM images than those measured using the SEM images. This might be explained by the fact that the samples were ultrasonicated in ethanol to facilitate the TEM observations, which led to small changes in the sizes of the sheet samples.

**Figure 3:**
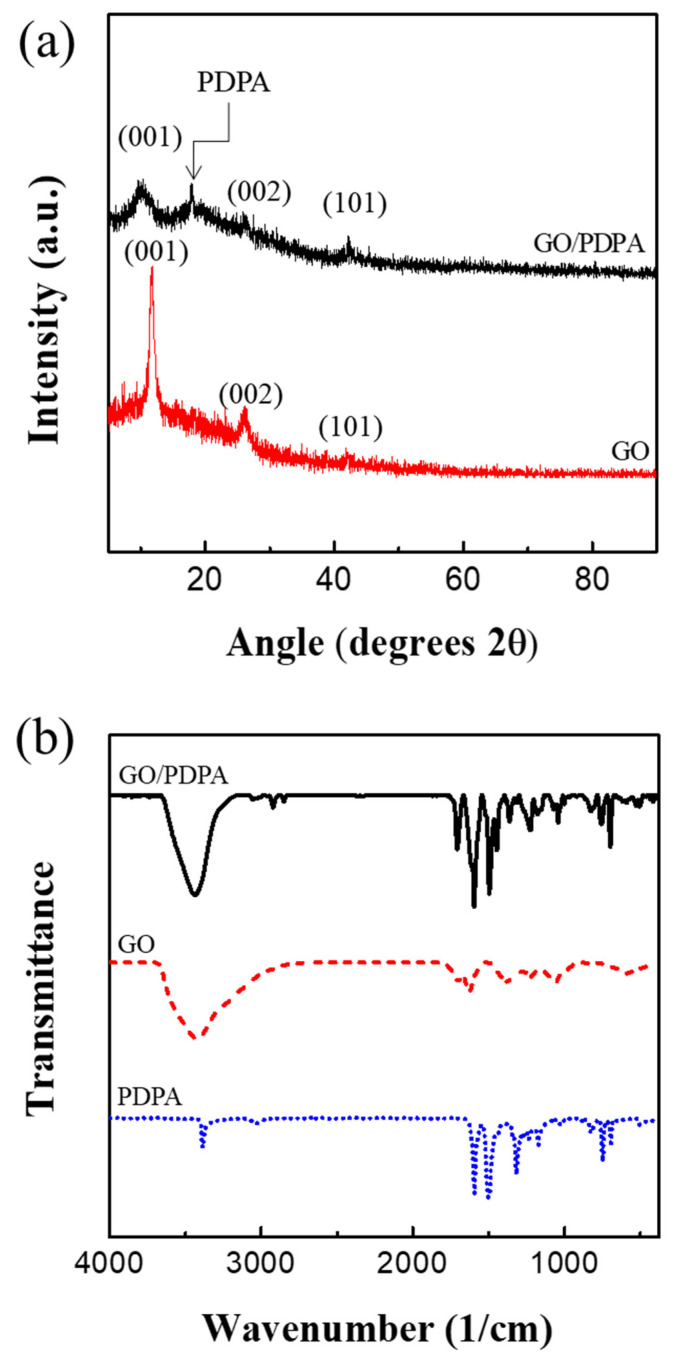
(**a**) X-ray diffraction profiles, (**b**) FTIR data of pure GO, PDPA, and GO/PDPA microsheet composites.

XRD and FTIR spectroscopy experiments were performed to characterize the crystalline structures and chemical components of the pure GO and the GO/PDPA microsheet composite (Figure 3). The GO sheets and GO/PDPA composite exhibit the typical (002) and (101) peaks at approximately 26.2° and 42.0°, respectively; the peaks are attributed to the graphitic framework. The GO sheets exhibit a sharp peak corresponding to the (001) plane at a 2θ of approximately 11.8°, whereas the GO/PDPA composite exhibits peaks corresponding to both the (001) GO base plane (2θ = 10.0°) and PDPA (2θ = 17.9°), which are shifted from those of the pure GO and PDPA [25,26]. The FTIR spectra of the GO, PDPA and GO/PDPA composite show high and broad absorbance signals at about 3430 cm^−1^ owing to the O–H stretching vibration. The neat GO sheets exhibit a distinctive signal at 1704 cm^−1^, originated to the C–O stretching vibration of carboxylic acid group. The peaks at 1616 and 1382 cm^−1^ are attributed to the O–H vibration in water and to CO–H groups in GO, respectively. Peaks corresponding to the C–O–C and C–O bonds are observed at 1228 and 1054 cm^−1^, respectively [14,27]. In the FTIR spectrum of GO/PDPA composites, not only the characteristic peaks of GO but also the characteristic peaks of PDPA are observed [19]. The GO/PDPA composite exhibits peaks at approximately 2921 and 833 cm^−1^, owing to the aromatic C–H stretching vibrations. The absorption signals at 1595, 1494, 1170, and 700 cm^−1^ are attributed to N–H bending vibrations, C=C stretching of the benzenoid ring, C–H in-plane bending of diphenoquinone, and C–H bending of the 1,4-substituted benzene ring, respectively [21,28]. These typical spectra are attributed to the contribution of the PDPA.

### 3.2. ER and Creep Properties

The ER fluid was formed using the semiconducting GO/PDPA microsheets (10 vol.%), suspended in silicone oil. The electrical conductivity of the GO/PDPA microsheets was 8.0 × 10^−8^ S/cm without dedoping, compared to 1.0 × 10^−6^ S/cm of the pure GO, which is within the ideal range for ER materials (10^−9^–10^−11^ S/cm) with regard to avoiding an electric short circuit [29].

**Figure 4:**
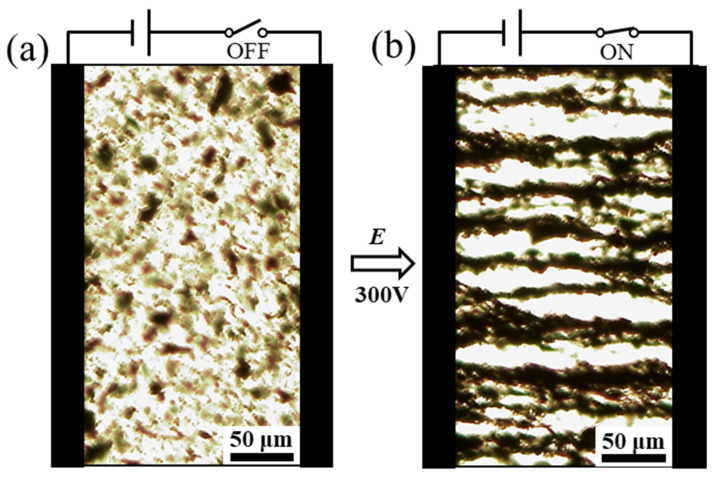
Optical microscopic images of GO/PDPA microsheet composites-based electrorheological (ER) fluid (10 vol.%) when the electric field was (**a**) off and (**b**) on (300 V).

**Figure 5:**
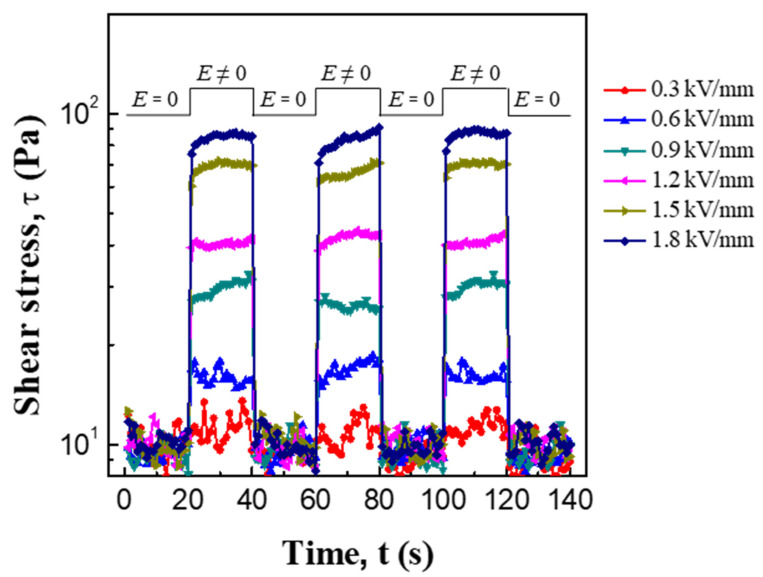
Shear stress vs. time of the GO/PDPA microsheet composites-based ER fluid influenced by alternating on–off state under different electric field at a fixed shear rate of 1.0 s^−1^.

Figure 4 presents the conducting property of the GO/PDPA microsheet-based ER suspension. Without an electric field, OM shows that the GO/PDPA sheets were randomly dispersed in the carrier oil, whereas the composites start to move to form flocculent fibrous microstructures along the electrical field direction at an applied direct-current voltage of 300 V. The response sensitivity and reversible behavior of the microsheet-based ER fluid are presented in Figure 5 (external square-type voltage pulse time: 20.0 s, constant shear rate: 1.0 s^−1^). The shear stress exhibits a square pulse response almost without hysteresis. The shear stresses instantaneously change at each turning point, and increase with the electrical field, similar to that measured using flow curves. This indicates that the GO/PDPA microsheet composite-based ER fluid is highly sensitive to and reversible under the input electrical field. As shown in Figure 5, under a stable electric field applied, most of the shear stress increases with time, which may be because the longer the electric field is applied, the stronger the chain structure is. In addition, during the shearing process at a given shear rate of 1.0 s^−1^, the chain structure is destroyed due to the hydrodynamic force and the process of reorganization due to electrostatic force occurs at all times. The decrease in shear stress may be due to the fact that the reorganized chain structure is not as stable as the original chain structure especially for lower electric fields.

**Figure 6:**
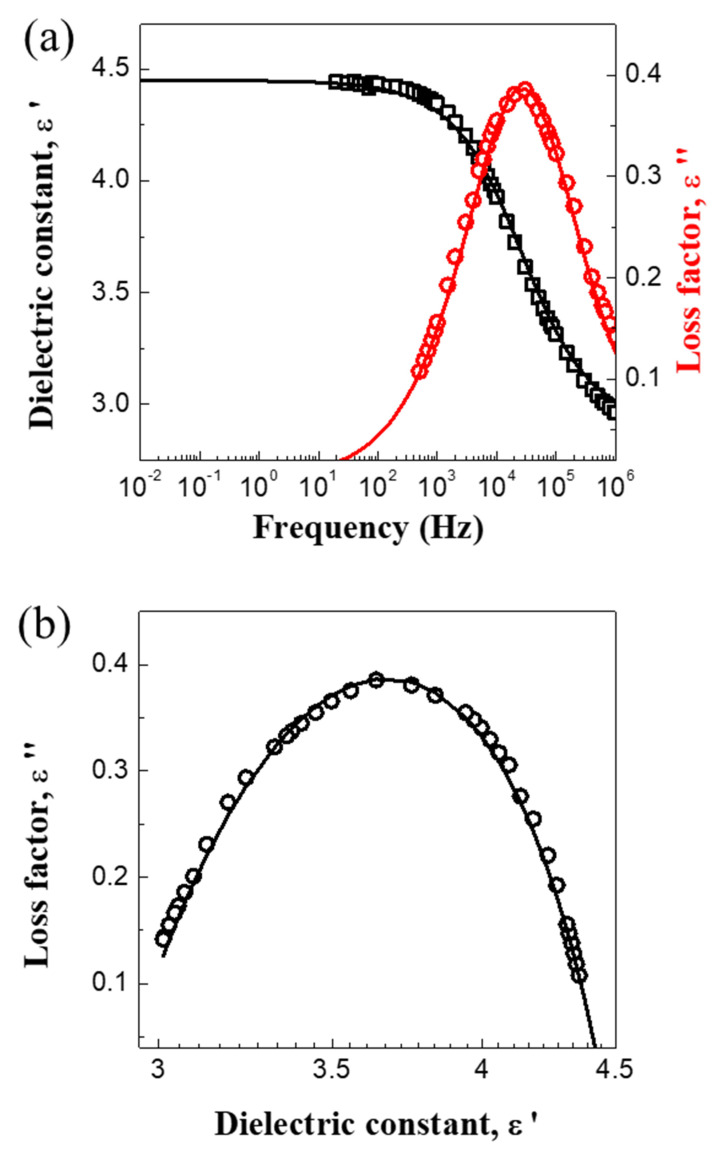
(**a**) Dielectric spectra (dielectric constant, ε′: black square; dielectric loss, ε″: red circle) and (**b**) Cole–Cole mode data of GO/PDPA microsheet composites-based ER fluid (10 vol.%).

Dielectric characteristics (dielectric constant ε′ and dielectric loss ε″) are one of the crucial factors that affect ER behavior. They were measured using the LCR meter. The ε′ and ε″ values of the composite-sheet-based ER fluid, as functions of the frequency, are shown in Figure 6a. Furthermore, ε′ and ε″ are related through the Cole–Cole formula (Figure 6b) [30,31],

(1) $$\varepsilon^{*}=\varepsilon^{\prime }+i\varepsilon^{\prime \prime }=\varepsilon_{\infty }+\frac{\Delta \varepsilon }{1+{\left(i\omega \lambda \right)}^{1-\alpha }}$$

where ε_0_ is the dielectric constant when the frequency goes to zero and ε_∞_ is the permittivity when the frequency tends to infinity. The difference Δε and λ represent the polarizability and polarization relaxation time of the ER suspension, respectively. The index 1 − α in the range of 0 to 1 implies the distribution broadness of the relaxation time. These parameters in the Cole–Cole equation are presented in Table 1.

**Figure 7:**
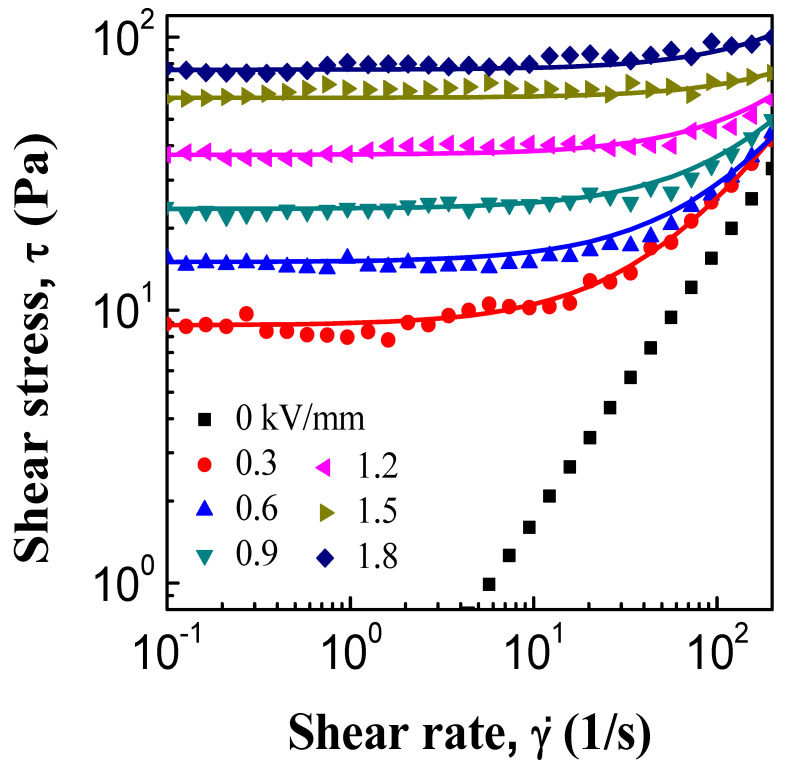
Shear stress vs. shear rate of the GO/PDPA microsheet composites-based ER fluid (10 vol.%) with different electric fields. The solid lines in graph are fitting from the Bingham fluid equation.

**Figure 8:**
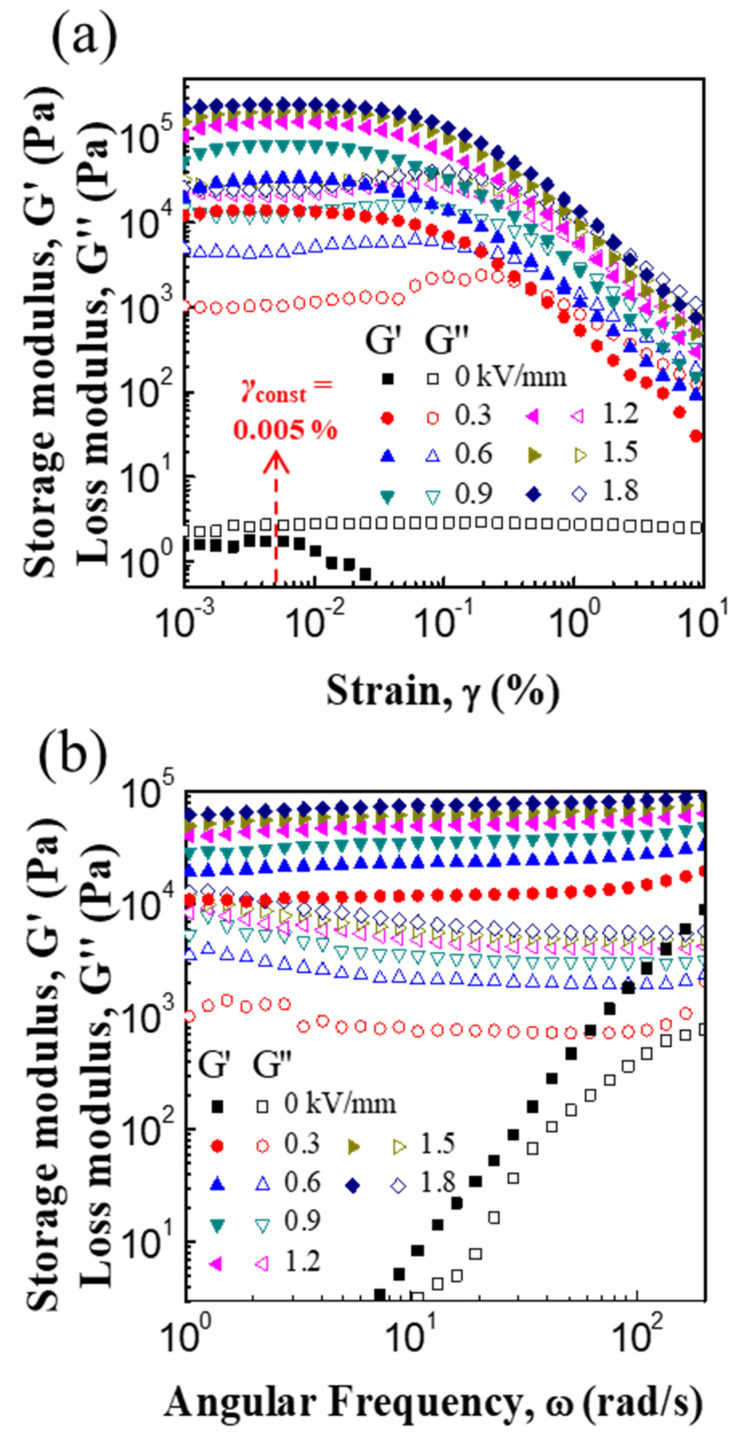
(**a**) Storage (closed) and loss (open) modulus vs. strain curves and (**b**) storage (closed) and loss (open) modulus vs. angular frequency curves of the GO/PDPA microsheet composites-based ER fluid (10 vol.%) with different electric fields.

For ER behaviors of the ER suspension, both steady shear and dynamic oscillation measurements were successively carried out using a rotational rheometer. Figure 7 shows typical flow curve of shear stress vs. shear rate at different electric field strengths. Without the electrical field, the ER fluid exhibits Newtonian fluid-like behaviors, including a linear increase in shear stress with an increase in shear rate, which is similar to the relationship between the dynamic modulus (storage or loss modulus) and angular frequency, observed in the oscillation test (Figure 8b). Under the electric field, the shear stress maintains relatively stable values regardless of the shear rate change at different applied electrical field strengths, consistent with the characteristics of Bingham fluids. The solid curves in Figure 7 are fitted using the Bingham fluid model (Equation (2)) [32]. The corresponding parameters are given in Table 2. *τ* and $\dot{\gamma }$ are the shear stress and shear rate, respectively, and τ_0_ is the yield stress.

(2) $$\tau =\tau_{0}+\eta \dot{\gamma }\;(\tau \geq \tau_{0})$$

The dynamic behaviors of the GO/PDPA microsheet-based ER fluid were characterized via amplitude sweep tests at a constant angular frequency of 6.28 rad/s (Figure 8a). Without the external electrical field, the storage modulus (G′) is lower than the loss modulus (G″), exhibiting more viscous nature of the ER fluid. With an induced electric field, the ER fluid gives elastic characteristics (higher G′) originating from the closely aligned microsheets, as shown in Figure 4 and Figure 5. Stepwise improvements in G′ and G″ are observed with an increase in the applied electric field strength. In the low-strain region, the ER fluids tend to form stable chain-like structures; this region is referred to as the linear viscoelastic (LVE) region. Within the γ_LVE_ region, γ was fixed to 0.005% to perform the frequency sweep measurement at angular frequencies of 1 to 200 rad/s, as presented in Figure 8b. G′ is higher than G″ with or without the input electrical field; thus, the elastic property precedes the viscous property. When E = 0, the higher volume percent (10 vol.%) and lower constant strain (0.005%) of the ER fluid promote the fluid’s elastic behavior.

**Figure 9:**
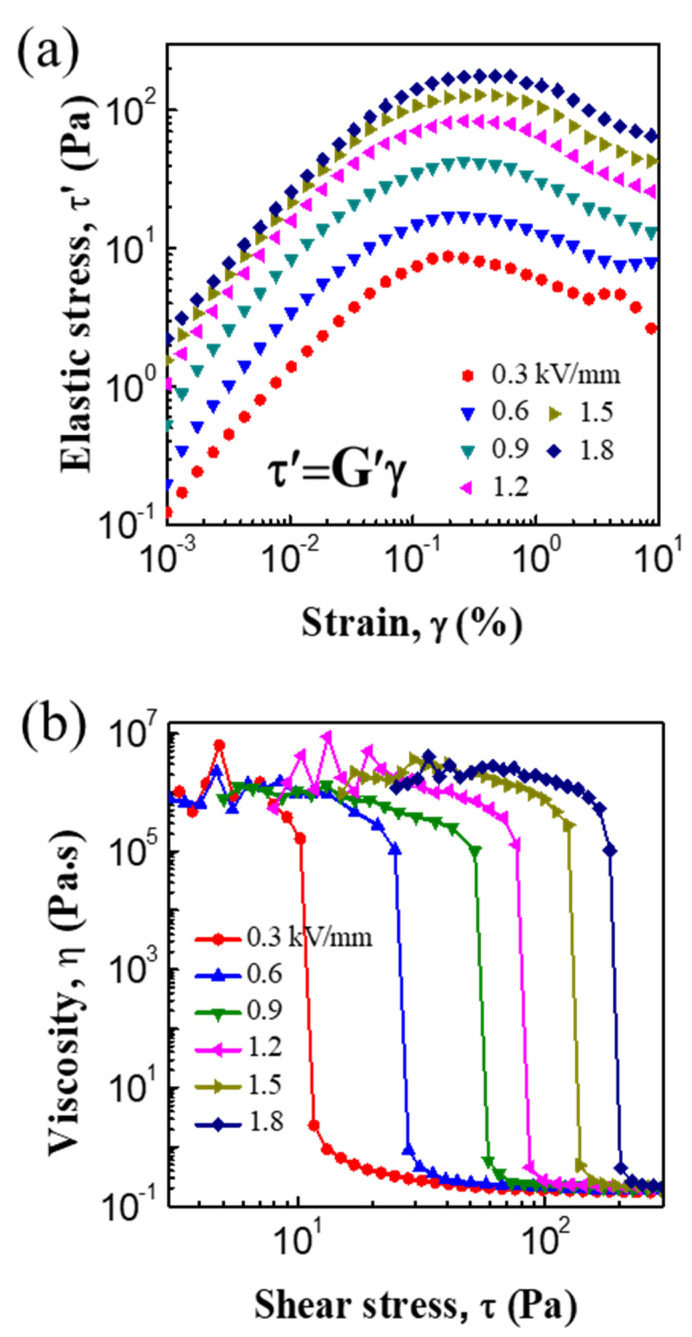
(**a**) Elastic stress vs. strain curves and (**b**) viscosity vs. shear stress curves of the GO/PDPA microsheet composites-based ER fluid (10 vol.%) with different electric fields.

**Figure 10:**
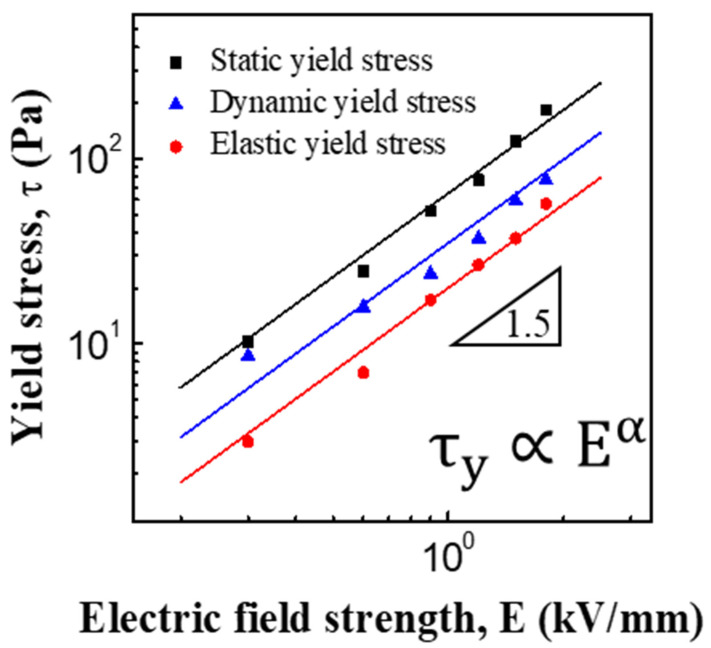
Yield stress vs. electric field strength curves of the GO/PDPA microsheet composites-based ER fluid (10 vol.%).

The elastic stress data in Figure 9a were calculated based on τ′ = G′γ, obtained from Figure 8a, where the elastic yield stress in the ER fluid is the maximum stress that enables complete restoration when the input electrical field is turned off; the maximum stress is obtained using the critical value, based on the changes in the slope. The static yield stress is the minimum shear stress necessary to ensure the fluidity of the ER fluid; this stress is observed using the controlled shear stress type (Figure 9b). At the various applied electrical field strengths, we select the stress corresponding to the sharp decrease in the critical shear viscosity as the static yield stress; this is presented in Figure 10 along with the elastic and dynamic yield stresses. The power-law relations between the three different yield stresses and electric field strength (E) (Equation (3)) (m = 1.5) obey the conduction model for ER fluids. Note that the slope close to 1.5 has been also observed for ER fluids based on not only GO composite [33,34] but also other composite particles [35].

*τ*_y_ ∝ *E*^m^ (3)

**Figure 11:**
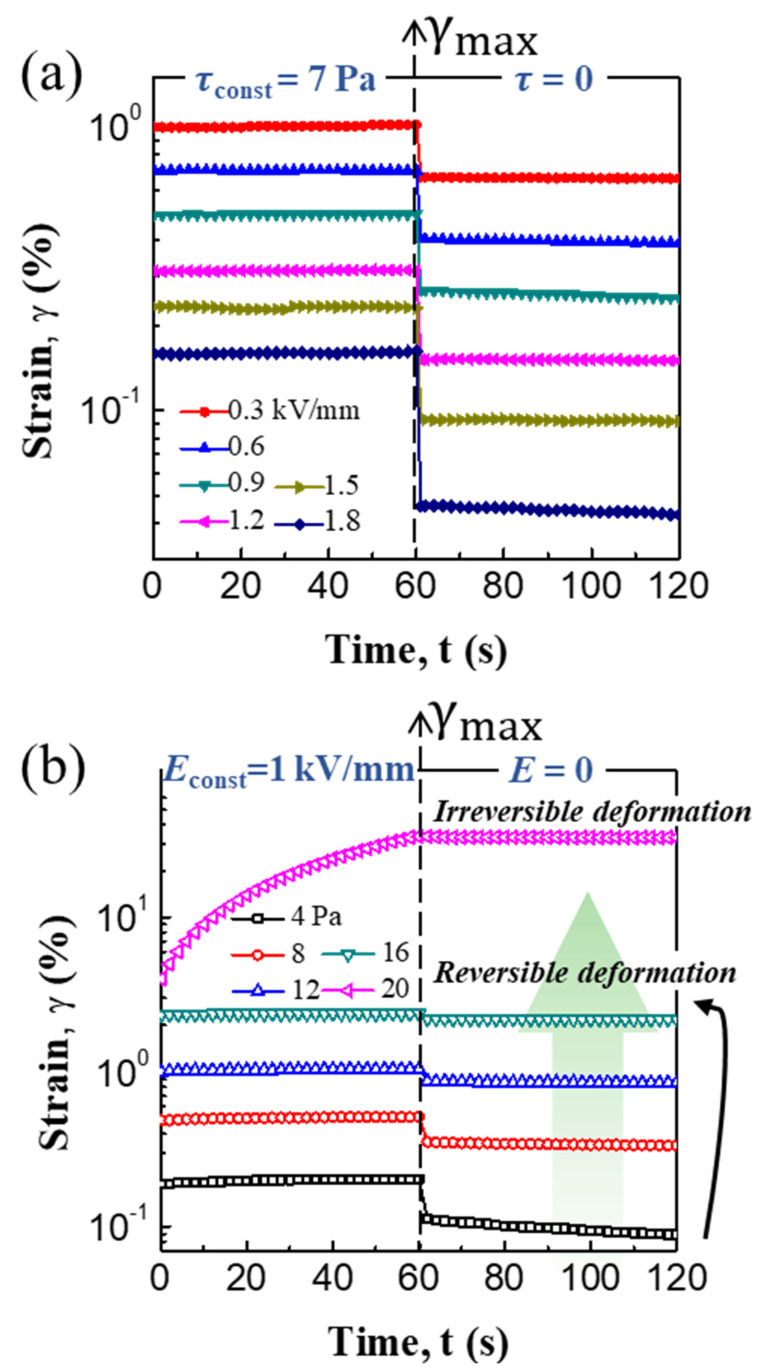
Strain vs. time under (**a**) constant stress and (**b**) constant electric field strength of GO/PDPA microsheet composites-based ER fluid (10 vol.%).

**Figure 12:**
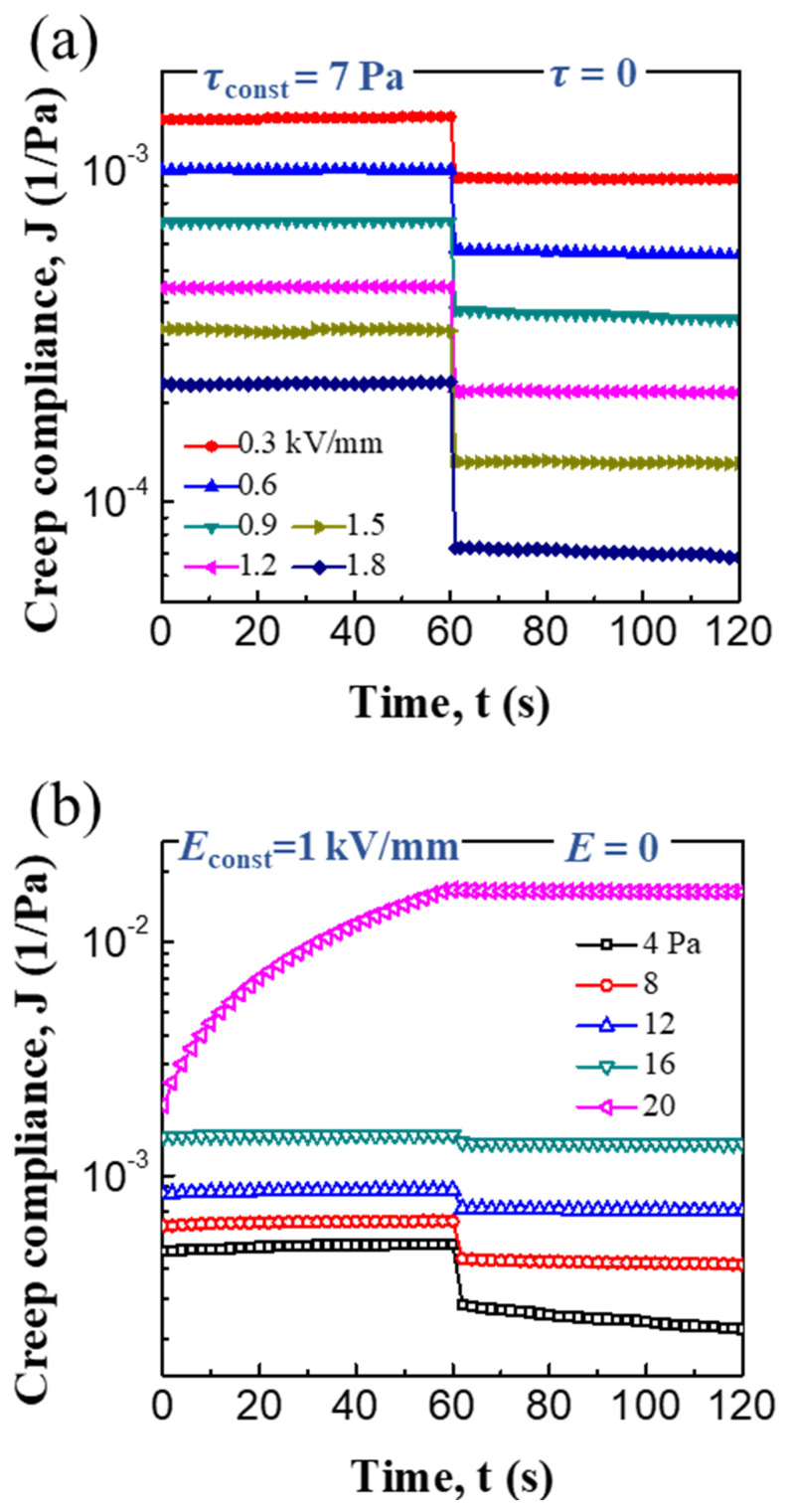
Creep compliance vs. time under (**a**) constant stress and (**b**) constant electric field strength of GO/PDPA microsheet composites-based ER fluid (10 vol.%).

**Figure 13:**
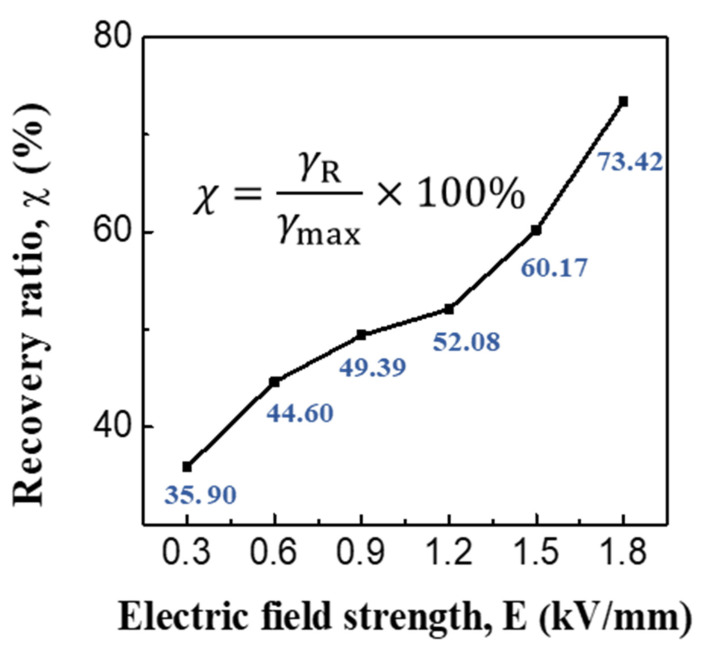
Recovery ratio vs. electric field strength of GO/PDPA microsheet composites-based ER fluid (10 vol.%).

**Figure 14:**
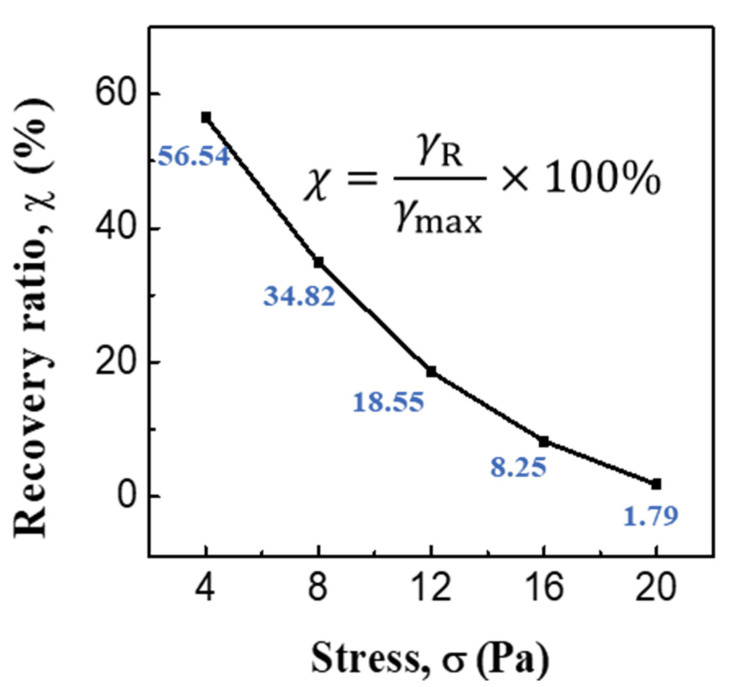
Recovery ratio vs. stress of GO/PDPA microsheet composites-based ER fluid (10 vol.%).

The creep and recovery behaviors of the GO/PDPA composite-based ER fluid were tested under a fixed shear stress and fixed electric field, as shown in Figure 11, Figure 12, Figure 13 and Figure 14. As a static test method, once a step change in stress is imposed during the creep test, immediate response of the strain is observed as a function of time. With this information, the creep compliance (J(t)), one of the indicators of creep characteristics, is defined as the ratio of the strain and shear stress (Equation (4)). For a constant shear stress of 7 Pa (Figure 11a, Figure 12a and Figure 13), the strain and creep compliance exhibit stepwise decreases with an increase in the applied electric field strength. This indicates that more stable chain-like structures are formed with the increase in electrical field to resist the constant shear stress. Notably, when the shear stress is applied or removed, the ER fluid immediately undergoes a strain or recovers from the strain without delay, which is different from the ER fluid creep phenomenon with time delay [36,37]. The creep test has been also performed for an ER elastomer [38]. The recovery ratio (χ) is the ratio of the recoverable strain (γ_R_) to the maximum strain (γ_max_) [39],

J(t) = (γ(t))/τ(4)

Χ = γ_R_/γ_max_ × 100% (5)

The recovery ratio gradually improves with an increased field strength; all cases correspond to reversible deformations at the fixed stress of 7 Pa. For the constant electric field strength of 1 kV/mm (Figure 11b, Figure 12b and Figure 14),Preparation of graphitic oxide. the strain and creep compliance steadily increase with the shear stress, up to 16 Pa. At a shear stress of 20 Pa, the ER fluid is immediately deformed and exhibits a continuously increasing strain delay until the stress is removed (it is almost irreversibly deformed). In other words, when E = 1 kV/mm, the shear stress of 20 Pa reaches the yield value, similar to the elastic yield stress in Figure 10. In addition, under a fixed electric field, with an increasing sustained shear stress, the recovery ratio gradually decreases until it approaches 0.

## 4. Conclusions

We successfully synthesized conducting composites of GO/PDPA microsheets, with a density lower than that of neat GO sheets, via in-situ oxidative polymerization. The PDPA uniformly adsorbed on the GO surface through π–π interactions. The electro-responsive ER behaviors of the GO/PDPA composites dispersed in silicone oil were analyzed using a rotational rheometer at various external electrical field strengths. In the steady shear tests, the GO/PDPA composite-based ER fluid showed a Newtonian fluid-like behavior without an electric field; these behaviors transformed into Bingham plastic characteristics under an input electric field. All three different types of yield stresses obtained from different rheological measurements showed a slope of 1.5, confirming its conductivity mechanism. Dielectric characteristics from the dielectric spectra were also found to fit well with Cole–Cole equation, being well correlated with the ER performance. According to dynamic oscillation tests, the storage modulus and loss modulus increased substantially under applied electric fields, which indicated the formation of chain-like structures in the ER fluid. Furthermore, from the creep and recovery tests under the applied electric field, it also showed distinctive solid-like properties in agreement with the observation from the steady shear and oscillation tests.

## Figures and Tables

**Table 1:** Optimum values of Cole–Cole formula for GO/PDPA sheet composites-based ER fluid.

Parameters	ε_0_	ε_∞_	Δ ε = ε_0_ − ε_∞_	λ (s)	α
value	4.45	2.91	1.54	6 × 10^−6^	0.41

**Table 2:** Optimal values for Bingham model on the basis of the flow curves for GO/PDPA composites-based ER fluid (10 vol.%).

**Model**	**Parameters**	**Electric Field Strength (kV/mm)**					
		0.3	0.6	0.9	1.2	1.5	1.8
Bingham	$\tau_{0}$	8.8	15.1	23.5	37.1	59.9	76.1
	*η*	0.17	0.14	0.13	0.12	0.07	0.13
